# Validity and Acceptability of Wearable Devices for Monitoring Step-Count and Activity Minutes Among People With Multiple Sclerosis

**DOI:** 10.3389/fresc.2021.737384

**Published:** 2022-01-11

**Authors:** Grace Lavelle, Meriel Norris, Julie Flemming, Jamie Harper, Joan Bradley, Helen Johnston, Jennifer Fortune, Andrea Stennett, Cherry Kilbride, Jennifer M. Ryan

**Affiliations:** ^1^Institute of Psychiatry, Psychology and Neuroscience, King's College London, London, United Kingdom; ^2^College of Health, Medicine and Life Sciences, Brunel University London, London, United Kingdom; ^3^The Hillingdon Hospitals, NHS Foundation Trust, Uxbridge, United Kingdom; ^4^Department of Public Health and Epidemiology, RCSI University of Medicine and Health Sciences, Dublin, Ireland; ^5^Wolfson Institute of Preventative Medicine, Queen Mary University of London, London, United Kingdom

**Keywords:** multiple sclerosis, wearable devices, physical activity, validity, acceptability, step-count

## Abstract

Multiple wearable devices that purport to measure physical activity are widely available to consumers. While they may support increases in physical activity among people with multiple sclerosis (MS) by providing feedback on their performance, there is little information about the validity and acceptability of these devices. Providing devices that are perceived as inaccurate and difficult to use may have negative consequences for people with MS, rather than supporting participation in physical activity. The aim of this study was, therefore, to assess the validity and acceptability of commercially available devices for monitoring step-count and activity time among people with MS. Nineteen ambulatory adults with MS [mean (SD) age 52.1 (11.9) years] participated in the study. Step-count was assessed using five commercially available devices (Fitbit Alta, Fitbit Zip, Garmin Vivofit 4, Yamax Digi Walker SW200, and Letscom monitor) and an activPAL3μ while completing nine everyday activities. Step-count was also manually counted. Time in light activity, moderate-to-vigorous activity, and total activity were measured during activities using an Actigraph GT3X accelerometer. Of the 19 participants who completed the validity study, fifteen of these people also wore the five commercially available devices for three consecutive days each, and participated in a semi-structured interview regarding their perception of the acceptability of the monitors. Mean percentage error for step-count ranged from 12.1% for the Yamax SW200 to −112.3% for the Letscom. Mean step-count as manually determined differed to mean step-count measured by the Fitbit Alta (*p* = 0.002), Garmin vivofit 4 (*p* < 0.001), Letscom (*p* < 0.001) and the research standard device, the activPAL3μ (*p* < 0.001). However, 95% limits of agreement were smallest for the activPAL3μ and largest for the Fitbit Alta. Median percentage error for activity minutes was 52.9% for the Letscom and 100% for the Garmin Vivofit 4 and Fitbit Alta compared to minutes in total activity. Three inductive themes were generated from participant accounts: *Interaction with device*; *The way the device looks and feels; Functionality*. In conclusion, commercially available devices demonstrated poor criterion validity when measuring step-count and activity time in people with MS. This negatively affected the acceptability of devices, with perceived inaccuracies causing distrust and frustration. Additional considerations when designing devices for people with MS include an appropriately sized and lit display and ease of attaching and charging devices.

## Introduction

Multiple sclerosis is a chronic, neurological condition affecting millions of people globally. It is estimated that a total of 2.8 million people live with a diagnosis of MS worldwide (35.9 per 100,000 population), with rising global prevalence rates observed since 2013 (1). Approximately 5,000 new cases of MS are diagnosed each year in the United Kingdom (UK), and it is more than two times as common in females than males (272 vs. 106 per 100,000 population) (2). A reduction in activity levels soon after diagnosis is common among people with MS, driving public health recommendations to help tackle these high levels of inactivity (3). Although physical activity may have several benefits for people with MS such as improved mental health, reduced fatigue, better walking performance, and lower mortality (4–7), many people with MS are inactive (8). Behavior change techniques, such as goal setting, providing feedback on performance, and self-monitoring of behavior, may support people with MS to increase physical activity (9). There is evidence from studies of the general population that monitoring physical activity alone results in an increase in physical activity (10). Multiple wearable devices that purport to measure physical activity are widely available to consumers. There is a large evidence base surrounding the validity of these devices to measure physical activity in the general population (11). Devices produced by Fitbit are, by far, the most frequently studied and appear to measure steps accurately, although validity may vary between devices (11). Gait deficits are a common feature of MS, and significant effects on gait even for those with relatively mild disability have been observed (12). This may affect the accuracy of these wearable devices when used by people with MS. However, few studies have specifically validated these devices in people with MS.

One study examined the criterion validity of five wearable devices and three smartphone applications for measuring steps among individuals with MS while walking on a treadmill (13). Devices included the Fitbit One and Fitbit Flex, Yamax SW200 Digi-walker, and Apple Health application. The Fitbit One demonstrated the best criterion validity for measuring steps, and measurement error was within 3% relative to manually counted steps (13), which is suggested as acceptable error (11). The Yamax SW200 and SW 401 were also compared against manually counted steps during treadmill walking in a group of adults with MS who could walk without an aid (14). Devices detected between 68.4 and 84.5% of observed steps during slow walking speeds but were more accurate during faster walking speeds, detecting between 95.6 and 100.5% of steps. Step-count from the Yamax SW200 over 7 days was also strongly correlated with step-count recorded by an Actigraph 7164 accelerometer (15, 16). Similarly, step-count recorded by the Fitbit Flex was strongly correlated with manually counted steps during overground walking and with steps recorded by an accelerometer over 7 days (17).

Although these studies provide some information on the validity of commercially available devices, most focus on one type of pedometer and not more recently developed wearables. Furthermore, criterion validity of these devices against manually counted steps was only assessed during walking. Despite being validated, adults with MS frequently perceived the Yamax SW200 to be inaccurate when monitoring their step-counts over 12 weeks (18). This suggests that, while these devices may be accurate for measuring step-count during walking in controlled environments, they are less accurate at measuring step-count during activities of daily life.

People with MS reported additional challenges with using the Yamax SW200 to monitor step-count, including difficulties attaching it, limited durability, and difficulties opening the device to view step-count on the digital display (18). These challenges resulted in frustration and had a negative impact on participants' motivation to increase step-count (18). These findings highlighted the importance of providing a device that is perceived as accurate and easy-to-use when asking individuals with MS to monitor physical activity. However, we were unable to identify any studies examining the acceptability of devices for monitoring physical activity among people with MS. Even if devices are valid, they will not be worn by people with MS and of little use for supporting physical activity behavior change, if they are not acceptable.

The aim of this study was, therefore, to assess the validity and acceptability of commercially available devices for monitoring step-count and activity time among people with MS.

## Materials and Methods

### Design

A mixed methods trial design was carefully chosen to collect both qualitative and quantitative perspectives of each of the five commercially available devices. Combining data on both the validity of each device, in addition to capturing the views of people with MS on the perceived acceptability of each device, allows for a broader interpretation of results, with more clinically meaningful applicability of findings, given the inclusion of the participant voice. Mixed-methods design has been recommended as best practice in health outcomes research to enhance scientific rigor and ensure a focus on patient-led priorities (19). This study was conducted in two phases. In the first phase, we examined the criterion validity of five devices that we considered commercially available by comparing step-count against manually counted steps. We considered monitors to be commercially available if they can be purchased by anyone in order to monitor daily activity and do not require purchasing additional software to process activity data. We also compared step-count from a device commonly used in research, the activPAL3μ, against manually counted step-count, to allow us to comment on the criterion validity of commercially available devices relative to a research standard device. We further compared activity minutes obtained from the commercially available devices to time in light, moderate-to-vigorous and total activity from the Actigraph GT3x+. We used a cross-sectional design for Phase 1 of this study. That is, data were collected on one occasion during a 3-h session at Brunel University London. Following completion of Phase 1, the participants were asked to participate in the second phase of the study, to examine the acceptability of the commercially available devices. In the second phase, the participants wore each monitor for 3 days of everyday use and participated in a short face-to-face semi-structured interview at the end of each 3-day period. This design emulates “real-life” usage compared to the limitations imposed in controlled laboratory environments such as with the validity components of this work. The directed focus on various factors of acceptability and the aim to summarize commonalities between the participants positions this phase within a subtle realist approach.

Ethical approval was provided by Brunel University London's Research Ethics Committee (REC) and the Health Research Authority and Health and Care Research West Scotland (REC reference 18/WS/0161). The participants provided written informed consent to participate in each phase of the study.

### Participants

The participants were recruited from an outpatient clinic at Hillingdon Hospital, MS support groups in the London Borough of Hillingdon, and a database of people with MS who previously consented to be contacted about research. Inclusion criteria were: a self-reported diagnosis of MS; over 18 years; relapse free for the past 3 months; able to independently walk with or without a walking aid within their home environment; free of unstable or acute medical conditions, e.g., unstable angina; and an ability to comprehend and follow all instructions relating to participation in the study. The participants were excluded if they were pregnant or participating in an alternative research study. The participants received a £20 voucher of their choice on completion of the study.

### Procedures

The participant's self-reported age, height, weight, Expanded Disability Status Scale (EDSS) score, patient determined disease steps (PDDS), type of MS, duration of MS, and self-selected walking speed were recorded. Walking speed was measured as the average of three trials of walking 10 m overground in a straight corridor. The participants completed activities using a walking aid if required.

#### Phase 1: Validity

The participants performed eight activities in a controlled environment at Brunel University London while wearing the seven activity devices simultaneously. These were all worn according to recommended placement areas, including the wrist, hip/waist, and mid-thigh. Where more than one monitor was placed in the same area, e.g., wrist, steps were taken to ensure good contact and to limit inappropriate movement of the device. The participants could choose not to perform an activity if they believed they were unable to complete it or if there were any safety concerns. The participants rested in a seated position between each activity. The activities are described in Table 1.

**Table 1:** Description of activities.

**Activity**	**Description**
Deskwork	Participants sat at a desk, browsed the internet, made a phonecall, and typed for ten minutes.
Elevator	Participants travelled up and down three floors in an elevator.
Washing and drying dishes	Participants washed and dried dishes at a sink for ten minutes
Stairs	Participants ascended and descended one flight of stairs.
Indoor walking	Participants walked indoors along a straight 10 m walkway at a self-selected speed.
Walking with obstacles	Participants walked indoors at a self-selected speed navigating around objects on the ground.
Outdoor walking	Participants walked outdoors along a predefined route on a path that included straight paths, bends, and stepping on and off curbs, at a self-selected speed.
Stationary cycling	Participants cycled for 10 minutes at a self-selected speed on an upright cycle ergometer.
Driving	Participants who arrived to their appointment by car drove a predefined loop of the university campus at 20 mph

##### Commercially Available Devices

We evaluated the following five commercially available devices: Fitbit Alta; Fitbit Zip; Garmin Vivofit 4; Yamax SW200 Digi-walker pedometer; and Letscom activity monitor. A description of each monitor is provided in Supplemental Material. We selected these devices as they vary in terms of (1) cost, (2) the type of data they collect, (3) how they are attached, (4) how data are displayed, and (5) the mode of charging. Supplementary Table 1 also gives a full breakdown of device selection in terms of cost (ranging from £19.75 to £100), functionality, and feature of each device. We ensured to cover a range of devices in terms of these specific attributes (including cost, PA monitoring, display, clock function, attachment, mode of charging, and prompts to move). These were identified as important factors by people with MS when choosing a device to monitor physical activity (18). The Fitbit Alta, Garmin Vivofit 4, and Letscom activity monitor are wrist-worn devices that measure step-count and active minutes. The participants wore these devices on their right, or a least-affected arm. Wristbands were tightened to prevent movement of the monitor during activity. The Fitbit Zip and Yamax SW200 Digi-walker pedometer are attached to the waistband of a person's clothes and monitor step-count. The participants wore these devices at their right, or a least-affected hip.

##### ActivPAL3μ

Steps were measured using the activPAL3μ activity monitor. The activPAL3μ is a small, lightweight device that is worn on the anterior aspect of the person's thigh. The participants wore the activPAL3μ on their right thigh. The activPAL3μ incorporates accelerometry and inclinometry data to provide information on step-count as well as the amount of time people spend on sedentary, upright, and ambulatory activities. Data were downloaded and processed using PAL software suite version 8.

##### Step Count

Steps were manually counted and measured during each activity using the five commercially available devices and the activPAL3μ. Steps were summed across each activity to provide total steps.

Step-count as displayed on the five commercially available devices at the start of the activity and at the end of the activity was recorded and subtracted to obtain steps measured during each activity. Additionally, the time that the activity started and stopped was recorded. Steps as measured by the activPAL3μ were calculated by extracting the corresponding time period from the activPAL3μ data and summing steps for this period. The participants were video-recorded, performing each activity, and steps for each activity were manually counted from video-recordings. Steps were counted by one individual. Accuracy of the step count by this rater was assessed by comparing it to steps counted by a second individual for 20% of the activities. Total steps across these activities were 1,802 for Rater 1 and 1,796 for Rater 2, an absolute difference of 6 steps or a difference of 0.3% relative to the first rater's step-count.

##### Activity Minutes

Activity minutes for the total period that activities were performed, from the start of the first activity to the end of the last activity, were identified from the display on the Fitbit Alta, Garmin Vivofit 4, and Letscom.

Minutes in light activity (LPA), minutes in moderate-to-vigorous activity (MVPA), and minutes in total activity were also obtained from the Actigraph GT3x+, which the participants wore on their right, or a least-affected side, at the hip during all activities. The Actigraph GT3x+ accelerometer is a small, lightweight triaxial accelerometer. Inbuilt sensors detect the magnitude of a person's acceleration in each plane, which is expressed as accelerometer counts per unit time. Data were collected in 1-s epochs.

Data were processed using the ActiLife 6 software. The time the first activity started and the time the last activity stopped was recorded. Accelerometer counts from the vertical axis were extracted for this time period. Accelerometer counts from the vertical axis only were used for data processing, because these were used to derive a cut point to classify MVPA in adults with MS (20). Minutes spent in MVPA were calculated by applying the cut point of 1,745 counts per minute, derived in a group of adults with MS, to the data (20). Light physical activity was identified as between 100 and 1,745 counts per minute. Minutes in total activity were calculated by summing time in LPA and time in MVPA.

#### Phase 2: Acceptability

The participants were asked to wear the five chosen commercially available devices over 15 days. They wore each monitor for three consecutive days. After the participants wore a monitor for 3 days, they participated in a brief semi-structured interview with the researcher regarding their perceptions of the acceptability of the monitor. A topic guide, developed from relevant literature and the aims of the study, was used to guide interviews. Questions included their experience of donning and doffing the monitor, process of using the device and seeing data, and perception of accuracy. The participants were then provided with the next monitor to wear for 3 days. The order in which the participants wore each monitor was randomized. After the participants wore the final monitor, they were asked an additional question about their preferred monitor and the comparable acceptability of the devices. Interviews were conducted in person at Brunel University London, in the participant's home, or in a location convenient for the participant. The interviews were audio-recorded and transcribed verbatim.

#### Data Analysis

The distribution of data was examined using appropriate graphs and tables. Mean, standard deviation, median, minimum, and maximum were used as appropriate to report participant characteristics, steps, and time in activity. We calculated the group percentage error for step-count as [(total steps from the device minus manually counted total steps)/manually counted total steps]^*^100 to allow comparison with other studies (11). We also report the number of the participants with a percentage error ≥5, ≥10, and ≥25%. Total steps from each device and manually counted steps were compared using paired *t*-tests. Bland-Altman plots were produced with 95% limits of agreement to compare agreement between each device and manually counted steps. We additionally calculated group percentage error for activity minutes for each device and compared activity minutes using Wilcoxon-signed rank tests. As it was unclear from the device manuals if activity minutes related to minutes in LPA, MVPA or LPA, and MVPA combined, we compared activity minutes from each device to minutes in LPA, MVPA, and total activity from the Actigraph GT3X. We did not calculate Bland-Altman plots for activity minutes as difference in activity minutes was not normally distributed. All analyses will be conducted using Stata version 14.0 (Statcorp, USA).

All interview recordings were transcribed and underwent framework analysis (21) by the same researcher. This method of analysis provides a clear audit trail of the analytical process, which enhances transparency. The technique involves five iterative stages of analysis; familiarization; identifying initial thematic framework through detailed line-by-line descriptive coding of the first five transcripts; labeling through which further minor adjustments were made to the framework; charting; mapping; and interpretation, following which significant themes can be presented. Labeling and charting included both deductive codes such as comfort of a device as well as inductive codes that arose from the participant narratives. Initial coding, labeling, and thematic development were discussed in detail with another researcher who was familiar with the data and who had independently coded three transcripts.

## Results

### Phase 1: Validity

Nineteen adults with MS were recruited to the study. Participant characteristics are presented in Table 2. The participants had a mean age of 52 years, ranging from 27 to 72 years. The majority were female with relapsing-remitting MS.

**Table 2:** Participant characteristics.

	***n* (%)**	**Mean (SD)**	**Median (range)**
Age, yr	19	52.1 (11.9)	55 (27, 72)
Women	13 (68.4)		
BMI, kg.m^−2^	19	29.4 (9.3)	26.3 (16.9, 52.2)
**Type of MS**			
Relapsing-remitting	16 (84.2)		
Secondary progressive	2 (10.5)		
Benign	1 (5.3)		
Relapse in past 3–12 months	5 (26.3)		
MS duration, yr	19	14.4 (11.8)	13 (0, 48)
**EDSS**			
1.0–4.0	10 (52.6)		
4.5–5.0	9 (47.4)		
**Mobility aid use over 5m**			
No aid	12 (63.2)		
Sticks or crutches	2 (10.5)		
Combination of sticks/crutches and wheelchair	5 (26.3)		1 (0, 5)
PDDS	19		1 (0, 5)
Walking speed, m/s	19	0.98 (0.36)	0.85 (0.64, 2.18)

One participant did not complete the walking with obstacles activity, two people did not complete the cycling activity, three people did not complete the outdoor walking activity, three people did not complete the stairs activity, and five people did not complete the driving activity. Additionally, outdoor walking was not video-recorded for one person. Steps for each activity as measured by each device and as manually counted are presented in Table 3.

**Table 3:** Description of steps measured by each device and manually counted for each activity.

	**Fitbit Alta**	**Fitbit Zip**	**Garmin Vivofit 4**	**Yamax SW200 Digi-Walker**	**Letscom**	**activPAL3μ**	**Manual count**
**Deskwork**							
Median (min, max)	0 (0, 19)	0 (0, 0)	0 (0, 15)	0 (0, 6)	0 (0, 11)	0 (0, 0)	0 (0, 0)
**Elevator**							
Mean (SD)	10.2 (6.7)	12.1 (6.5)	11.6 (10.8)	12.7 (8.4)	−1.3 (5.7)	6.1 (3.0)	22.9 (7.6)
Median (min, max)	11 (0, 25)	14 (0, 21)	11 (0, 30)	14 (0, 21)	0 (-25, 0)	5.5 (3, 14)	21 (14, 48)
**Washing dishes**							
Mean (SD)	169.8 (125.6)	15.4 (13.4)	143.4 (71.6)	19.0 (13.4)	406.6 (255.2)	7.4 (4.5)	54.1 (29.0)
Median (min, max)	130 (24, 504)	13 (6, 67)	130 (59, 315)	15 (4, 57)	307 (0, 840)	5.5 (2, 19)	40 (20, 131)
**Stairs^a^**							
Mean (SD)	26.2 (11.3)	28.4 (8.2)	28.3 (15.3)	26.1 (8.2)	18.8 (19.6)	13.9 (2.8)	32.4 (4.6)
Median (min, max)	30 (0, 41)	31 (0, 36)	34 (0, 45)	31 (0, 36)	16 (0, 43)	14 (10, 20)	31.5 (27, 45)
**Indoor walking**							
Mean (SD)	32.6 (26.2)	37.0 (7.2)	37.5 (18.1)	36.6 (8.8)	32.3 (23.3)	18.4 (6.5)	40.3 (12.7)
Median (min, max)	33 (-62, 76)	36 (18, 56)	39 (0, 78)	36 (25, 67)	37 (0, 86)	16 (13, 35)	36 (28, 77)
**Outdoor walking^b^**							
Mean (SD)	274.4 (72.9)	254.4 (50.2)	280.8 (109.4)	182.6 (99.4)	271.4 (81.7)	137.5 (45.7)	304.1 (187.0)
Median (min, max)	258.5 (219, 530)	256 (119, 374)	263 (144, 667)	236 (0, 286)	265.5 (152, 552)	128 (107, 287)	262 (218, 976)
**Cycling^c^**							
Median (min, max)	81 (−9, 642)	0 (0, 544)	0 (0, 777)	1 (0, 154)	0 (0, 480)	0 (0, 0)	0 (0, 0)
**Driving^d^**							
Median (min, max)	29.5 (0, 144)	2 (0, 14)	112 (19, 236)	40 (1, 66)	0 (-29, 99)	0 (0, 0)	0 (0, 0)
**Obstacles^e^**							
Mean (SD)	47.9 (25.1)	47.3 (9.3)	61.3 (28.6)	42.1 (14.7)	57.0 (26.7)	26.2 (10.3)	61.1 (44.8)
Median (min, max)	43 (11, 123)	49.5 (19, 59)	55 (0, 130)	42.5 (16, 80)	48.5 (0, 120)	23, (19, 56)	48 (8, 222)
**Total**							
Mean (SD)	745.3 (357.8)	448.6 (249.7)	693.5 (289.6)	323.1 (118.7)	832.5 (314.7)	179.6 (83.7)	442.5 (269.6)
Median (min, max)	745 (147, 1425)	409 (83, 956)	730 (190, 1259)	365 (91,491)	965 (316, 1378)	190.5 (45, 428)	450 (101, 1442)

*n = 19 for all devices except for activPAL3μ (n = 16) unless stated otherwise*;

a *n = 16 for all devices except for activPAL3μ (n = 13)*;

b *n = 16 for all devices except for activPAL3μ (n = 13) and manual count (n = 15)*;

c *n = 17 for all devices except for activPAL3μ (n = 15)*;

d *n = 14 for all devices except for activPAL3μ (n = 13)*;

e *n = 18 for all devices except for activPAL3μ (n = 15)*.

Percentage errors for each device are reported in Table 4. Percentage error was smallest for the Yamax SW200, although the Fitbit Zip had a similar percentage error. The Yamax SW200 and Fitbit Zip also had the fewest number of people with an error of >25%. Although the error for the research standard device, the activPAL3μ, was >25% for all the participants, the range was narrowest for the activPAL3μ. All devices except for the research standard device, the activPAL3μ, both overestimated and underestimated steps. The research standard device, the activPAL3μ, consistently underestimated steps by between 54 and 70%.

**Table 4:** Percentage error between visually counted steps and steps from each monitor.

**Device**	**% Error, mean (95% CI)^a^**	**% Error, minimum, maximum (range)**	**≥ 5% Error, *n* (%)**	**≥10% Error, *n* (%)**	**≥25% Error, *n* (%)**
Fitbit Alta	−80.6 (−113.0, −48.3)	−216.7, 48.3 (265.0)	19 (100)	18 (95)	18 (95)
Fitbit Zip	−14.9 (−47.0, 17.2)	−151.6, 82.8 (234.4)	17 (89)	14 (74)	8 (42)
Garmin Vivofit 4	−67.6 (−90.9, −44.2)	−168.4, 28.8 (197.2)	18 (95)	18 (95)	16 (84)
Yamax SW200	12.1 (−5.2, 29.4)	−79.5, 93.7 (173.2)	16 (84)	13 (68)	7 (37)
Letscom	−112.3 (−148,8, −75.9)	−318.8, 32.7 (351.5)	19 (100)	19 (100)	18 (95)
activPAL3μ^b^	59.0 (56.6, 61.3)	54.2, 70.3 (16.1)	19 (100)	19 (100)	19 (100)

a *Positive value indicates the device underestimates steps in comparison to manual count*.

b *n = 16*.

According to manually counted steps, all the participants had zero steps during deskwork, cycling, and driving. The research standard device, the activPAL3μ, also recorded zero steps for all the participants during these activities. The Fitbit Alta recorded >0 steps for four participants (21.1%) during deskwork, 13 participants (76.5%) during cycling, and 13 participants (92.9%) during driving. The Fitbit Zip recorded zero steps during deskwork. However, it recorded >0 counts for five participants (29.4%) during cycling and seven participants (50%) during driving. The Garmin Vivofit 4 recorded >0 steps for six participants (31.6%) during deskwork, eight participants (47.1%) during cycling, and all participants (*n* = 14) during driving. The Yamax SW200 Digi-walker pedometer recorded >0 steps for seven participants (36.8%) during deskwork, nine participants (52.9%) during cycling, and all the participants (*n* = 14) during driving. The Letscom monitor recorded >0 steps for one participant (5.3%) during deskwork, five participants (29.4%) during cycling, and five participants (35.7%) during driving.

The mean difference in total steps between manually counted steps and each monitor is described in Table 5. There was evidence that mean total steps differed between manually counted steps and the Fitbit Alta (*p* = 0.002), Garmin Vivofit 4 (*p* < 0.001), Letscom (*p* < 0.001), and research standard device, the activPAL3μ (*p* < 0.001). However, 95% limits of agreement were narrowest for the research standard device, the activPAL3μ, followed by the Garmin Vivofit 4 (Table 5). Limits of agreement were largest for the Fitbit Alta. However, they were similar for the Fitbit Zip.

**Table 5:** Difference and agreement between visually counted steps and steps from each monitor.

**Monitor**	**Mean difference (95% CI), steps**	***p*-value**	**95% Limits of agreement, steps**
Fitbit Alta	−302.84 (-483.32, −122.36)	0.002	−1036.8, 431.1
Fitbit Zip	−6.16 (−181.05, 168.73)	0.942	−717.4, 705.0
Garmin Vivofit 4	−251.05 (-375.15, −126.95)	<0.001	−755.7, 253.6
Yamax SW200 Digi-walker pedometer	119.37 (−32.46, 271.19)	0.116	−498.0, 736.8
Letscom	−390.00 (−541.65, −238.35)	<0.001	−1006.7, 226.7
activPAL3μ^a^	277.31 (165.72, 388.90)	<0.001	−133.1, 687.8

*Mean difference calculated as manually counted total steps minus monitor total steps*.

a *n = 16*.

Activity minutes reported by the Letscom, Fitbit Alta, and Garmin Vivofit 4, and minutes in LPA, MVPA, and total activity measured by the Actigraph GT3x+ are provided in Table 6. The Fitbit Alta recorded 0 activity minutes for 17 participants (89.5%), and the Garmin Vivofit 4 recorded 0 activity minutes for all 19 participants (100%). The Letscom did not record 0 activity minutes for any participant. No participant had 0 min in LPA or total activity as measured by the Actigraph GT3X. Six participants (33.3%) had 0 min in MVPA. Of these participants, all six had 0 activity minutes recorded by the Garmin Vivofit 4, five had 0 min recorded by the Fitbit Alta, and none had 0 min recorded by the Letscom. In comparison to min in total activity, median error was 52.9% for the Letscom and 100% for the Garmin Vivofit 4 and Fitbit Alta (Table 7). In comparison to minutes in LPA, median error was 48.6% for the Letscom and 100 for the Garmin Vivofit 4 and Fitbit Alta. There was a difference between activity minutes from each device and minutes in LPA and total activity (Table 7). There was also a difference between activity minutes and minutes in MVPA for the Letscom and Garmin Vivofit 4, but not for the Fitbit Alta (*p* = 0.052).

**Table 6:** Description of activity time measured by Fitbit Alta, Garmin Vivofit 4, Letscom, and Actigraph GT3x.

**Monitor**		**Activity time**		**Light activity**		**Moderate-to-vigorous activity**		**Total activity**	
		**Mean (SD)**	**Median (min, max)**	**Mean (SD)**	**Median (min, max)**	**Mean (SD)**	**Median (min, max)**	**Mean (SD)**	**Median (min, max)**
Fitbit Alta	19	3.0 (10.2)	0 (0, 43)	n/a	n/a	n/a	n/a	n/a	n/a
Garmin Vivofit 4	19	0 (0)	0 (0, 0)	n/a	n/a	n/a	n/a	n/a	n/a
Letscom	18	18.7 (5.3)	18.5 (8, 31)	n/a	n/a	n/a	n/a	n/a	n/a
Actigraph GT3x+	18	n/a	n/a	34.6 (11.7)	31.5 (17, 56)	3.2 (3.2)	3 (0, 11)	37.7 (12.1)	35 (17, 57)

**Table 7:** Percentage error, mean difference, and 95% limits of agreement between total activity measured by Actigraph GT3x and activity minutes from each monitor.

**Device**	***n***	**% error, Median^a^**	**% error, Minimum, maximum (range)**	**≥ 5% error, *n* (%)**	**≥ 10% error, *n* (%)**	**≥ 25% error, *n* (%)**	***p*-value^b^**
**Total activity**							
Letscom	17	52.9	5.6, 65.1 (59.6)	17 (100)	16 (94.1)	15 (88.2)	<0.001
Fitbit Alta	18	100.0	−38.7, 100.0 (138.7)	18 (100)	18 (100)	18 (100)	<0.001
Garmin Vivofit 4	18	100.0	100.0, 100.0 (0.0)	18 (100)	18 (100)	18 (100)	<0.001
**Light physical activity**							
Letscom	17	48.6	5.3, 63.3 (58.1)	17 (100)	15 (88.2)	14 (82.3)	<0.001
Fitbit Alta	18	100.0	−38.7, 100.0 (138.7)	18 (100)	18 (100)	18 (100)	<0.001
Garmin Vivofit 4	18	100.0	100.0, 100.0 (0.0)	18 (100)	18 (100)	18 (100)	<0.001
**Moderate to vigorous physical activity^c^**							
Letscom		n/a	n/a	n/a	n/a	n/a	<0.001
Fitbit Alta		n/a	n/a	n/a	n/a	n/a	0.052
Garmin Vivofit 4		n/a	n/a	n/a	n/a	n/a	<0.001

a *Positive value indicates the device underestimates steps in comparison to Actigraph GT3x*

b *p value obtained from Wilcoxon signed rank test comparing activity time from each device to Actigraph GT3X*.

c *Unable to calculate percentage error because some people had 0 minutes of moderate-to-vigorous physical activity as measured by the Actigraph GT3X*.

### Phase 2: Acceptability

All 19 participants were invited to participate in Phase 2 of the study. Fifteen agreed and provided written informed consent to participate. Three inductive themes were generated from participant accounts: *Interaction with device*; *The way the device looks and feels; Functionality*. These are described below, illustrated through anonymised quotations.

#### Interactions With Device

This theme focuses on the accessibility of the device for individual use in which the qualities of the display and ease of data retrieval and charging were the key. A frequently noted feature was the importance of visibility with the participants preferring screens that were well-sized (noted 10 times), well-lit (five references), and easy to read (14 references). The Letscom received the highest number of positive comments in this regard:

“*Display Is Clear, White on Black. Even in low Light, Is Easy to see.” (Letscom-Participant 14)*.

“*Nice big time and date, heart rate easy to read.” (Letscom-Participant 13)*.

The importance of these features was noted by their absence or inadequacy (13 references to inadequate size, 13 references to inadequate or absent lighting, and 19 references to excessive complexity of display). The GarminVivofit 4 received 32 concerns, nearly double the number of any other device.

“*Display is too small and not clear in different things to monitor. I don't understand the icons. It's not bright enough.” (Garmin-Participant 2)*.

“*Don't like it. Small and compact… Screen too dark to read and not very clear with icons - difficult with vision problems.” (Garmin-Participant 3)*.

Of note here is the specific reference to potential visual problems, which are common in people with MS.

All the participants tried to retrieve data from the devices; nine people tried to retrieve data from the associated applications, and 13 people from the device itself. While 12 people noted that they had no difficulty with retrieving data, with the Letscom most commonly being noted as the easiest with eight references, challenges were raised with synching data between devices. The GarminVivofit 4 received the majority of negative comments (six references). This resulted in frustration and an inability to monitor their activity appropriately.

“*syncing with phone is a nightmare. Does not self-sync and then does not record. Really aggravating to use. Needs auto-synch.” (Garmin-Participant 12)*.

“*seems to underestimate steps in the app - what was on the watch and what was on the app was different (2,000 steps). So syncing issue.” (Garmin-Participant 2)*.

Further frustration came when charging the device was problematic. Only two of the devices used in this study required to be charged, the Letscom and Fitbit Alta. The majority of the participants (10 out of 11) that charged the Alta described the process as simple. In comparison, a large proportion of the participants who attempted to charge the Letsom (9 out of 10 participants) commented that this was a difficult process. The participant accounts detailed that this was due to the stiffness of the device and concern over causing damage. In response to the question “did you charge the Letscom?,” one participant stated:

“*yes, with major problems - nightmare. Need to wiggle and concerned with breaking charging port. Not easy at all. Husband also struggled. Will be a problem for anyone with hand problems.” (Letscom-Participant 3)*.

The reference to the challenge for people with dexterity issues is particularly relevant, given the prevalence of alterations in sensation and fine motor control in people with MS.

#### The Way the Device Looks and Feels

This theme draws together participant responses to both the aesthetic and comfort of the device. The participants were asked to score the comfort of devices out of 10, alongside feedback. The aesthetic of the device was not a component included in the topic guide but was noted as an important factor by many participants.

The Fitbit Zip, Fitbit Alta, and Yamax SW200 Digi-walker pedometer were generally seen as the most comfortable devices (mean scores of 9.3, 8.7, and 8.1, respectively). In the case of the Zip and Yamax SW200 Digi-walker pedometer, this appeared to relate to their positioning on the hip as the participants reported being fundamentally unaware of its presence once donned:

“*Very comfortable 8/10. Didn't know I had it on.” (Fitbit Zip-Participant 1)*.

“*10/10 - comfortable, tight fit, and stayed put, forget you‘re wearing it” (Fitbit Zip-Participant 10)*.

All devices had some less positive comments on comfort. These related to the device catching on clothes or objects (Fitbit Zip, Letscom, and Yamax SW200 Digi-walker pedometer), the material of the product (all monitors), and irritation of the skin (Fitbit Alta, GarminVivofit, 4 and Letscom). However, these comments were limited, and, overall, all devices were considered acceptably comfortable (mean for each device range 7.1–9.3/10).

Many participants emphasized the importance of the aesthetic of the device (22 comments), requiring it not just to be fashionable, but to look sleek, modern, and up-to-date**:**

“*It is comfortable and looks fashionable” (Letscom-Participant 6)*.

“*Also liked having a watch on the screen. Neat and tidy little thing” (Fitbit Alta-Participant 7)*.

In contrast, devices were rejected if they disturbed the look of an outfit, usually through unwanted hip bulges. In general, female participants appeared to hold more importance than male in the aesthetic and design of the device (20/22 comments were from female participants). Additionally, several female participants commented on external judgement of the aesthetic, with colleagues or partners contributing to their positive or negative opinions of the device.

#### Functionality

When considering the functionality of the devices, the participants highlighted several relevant features, including perceived accuracy, the capacity of the device to encourage more physical activity, and the ease of donning and doffing the device and its stability once on.

The perceived accuracy of the data collected by the device was pivotal to its acceptability. These perceptions were based on the participants' self-monitored activity levels, and, in some cases, compared directly to devices already owned. While a range of positive comments were noted (32 comments across different devices), concerns predominated (45 comments). These were particularly noted with the YamaxSW200 Digi-walker pedometer (15 references), but also the Garmin Vivofit 4 (9 references) and Letscom (nine references). Perceived inaccuracy was an unacceptable quality in the device and led them to have strong negative views:

“*If someone bought this thinking it was gonna help, especially someone with a medical condition, and it says, oh you've done these many steps, that's probably not great because if it's not consistent, then you can‘t see a consistent change…..….It could be misleading if someone was really trying to look after their health and fitness or improve their step count, and that makes me kind of cross!” (Letscom-Participant 10)*.

“*Make a note, it‘s lying. I discovered, ha haha. I‘m still sitting on the sofa here, and by moving back is one step. Liar, you‘re a liar. You can‘t even trust a funny ticky walk I had anymore. You are out of my life; I don't want you in my life. I thought I could love you, but I don't.” (Yamax-Participant 6)*.

As noted in the first quote, monitoring activity was expected to help the individual and over 50% of the participants discussed motivational elements of the device as unprompted comments. Positive motivational elements in the devices such as the ability to set goals, and prompts to move were valued.

“*Yeah. Handy to see how active you are. Like to see steps as know goal is 10,000.” (Fitbit Alta-Participant 11)*.

“*Gets a sense of achievement by doing more.” (Fitbit Alta-Participant 6)*.

The different devices had varying levels of monitoring and output. Accounts from the participants highlighted that levels of functionality of the device and accompanying app were a factor of acceptability. Where some were happy with basic functionality provided; others described wanting and liking a more holistic approach:

“*Good info on the App-nutrition, trends, drinking water. After looking at how much water I should be drinking, I bought myself a water cup with measurements on it.” (Fitbit Zip-Participant 13)*.

“*Would prefer something that measures heart rate, as can see patterns in my stress levels. From experience, I use heart rate levels on my own device to monitor when I need to rest.” (Garmin-Participant 10)*.

In contrast, several participants also described too much functionality as a negative component of a device, which, at times, could become off-putting or even de-motivating.

The ability to don and doff the device and ease of doing so were direct questions to all the participants for all devices. As with the ability to charge the device, the responses illuminate challenges in the required levels of dexterity, sensory integration, and strength:

“*Trying to get it onto the trousers was fiddly. Pins and needles make it difficult. I put it on before the trousers went on.” (Fitbit Zip-Participant 7)*.

“*It was stiff which was quite tricky - rubberised case, the spring is too strong. I needed both hands.” (Fitbit Zip-Participant 3)*.

“*Hated it. Very difficult. Can‘t do the catch, its fiddly.” (Yamax-Participant 13)*.

Problems in this regard were almost exclusively noted with the devices that attached to clothes at a hip level.

## Discussion

This study aimed to assess the validity and acceptability of commercially available devices for monitoring step-count and activity time among people with MS. Of the commercially available monitors, the Garmin Vivofit 4 demonstrated the best agreement with manually counted steps. However, the mean step-count from the Garmin Vivofit differed to the mean manually counted steps, and the limits of agreement were wide, suggesting poor agreement and likely inaccurate measurement of step-count for individuals with MS. The research standard device, the activPAL3μ, performed best at measuring step-count in individuals with MS. All monitors, including the activPAL3μ, provided poor estimates of time in activity.

Percentage error was smallest for the Yamax SW200 and largest for the Letscom when compared to manually counted steps. However, percentage error for all monitors was high in comparison to that reported in a review of the validity of commercially available wearable devices among adults without mobility limitations and/or chronic diseases (133 studies) and without these conditions (36 studies) (11). Of 805 comparisons between devices and a criterion measure, 45.2% were within ± 3% measurement error, 42.7% were below −3% measurement error, and 12.1% were above 3% measurement error (11). This suggests that these devices perform particularly poorly in adults with MS. However, differences could also be due to the procedure used to validate the device and the type of the device.

Of the devices examined, only the Yamax SW200 had been validated against manually counted steps in people with MS. The Yamax SW200 detected between 68.4 and 100.5% of steps during treadmill walking at various speeds (14). Another study found a similar percentage error for the Yamax SW200 (8.5% compared to 12.1% in this study) (13). However, the proportion of the participants with ≥5, ≥10, and ≥25% error was much higher in our study than in the previous study (84 vs. 24%; 68 vs. 20%; and 37 vs. 11%) (13). This may be because we assessed criterion validity during a range of activities of daily living, while the previous study assessed criterion validity during two 500-m walking trials at a comfortable speed on a treadmill. Although these specific devices have not been evaluated in people with MS, four wearable motion sensors and three smartphone applications were evaluated in addition to the Yamax SW200 during treadmill walking (13). Percentage error for other devices ranged from 1.9 to 14.2% (13), with the Fitbit One demonstrating best absolute and relative precision, and fewest participants with ≥5% error (13). The higher percentage error we observed in this study for all monitors may be because they were evaluated during a range of activities and not just walking. Performance of the device likely differs, depending on the activity performed, which highlights the importance of assessing validity during everyday activities in order for findings to be more applicable to free-living conditions. One study compared daily step-count measured from the Fitbit Flex against daily step-count from an Actigraph accelerometer and observed a strong correlation but a difference in mean daily step-count between devices (17). Similarly, strong correlations were observed between the Yamax SW200 and Actigraph 7164 in free-living settings (15, 16). Correlation, however, does not equate to agreement (22).

All devices in this study had at least a 5% error for 84% or more of the participants compared to manually counted steps, with the research standard device, the activPAL3μ, having at least 25% error for all the participants. However, examining percentage error alone disguises the variability in individual error. The range of error for the research standard device, the activPAL3μ, was much smaller than for any other monitor at 16.1%. The range of error for other monitors was between 173.2% for the Yamax SW200 and 351.5% for the Letscom. This is reflected in the 95% limits of agreement, with the research standard device, the activPAL3μ, having the narrowest limits of agreement despite the mean step-count recorded by the activPAL3μ, being different to mean manually counted steps. The research standard device, the activPAL3μ, also consistently underestimated step-count, while other monitors under- and overestimated step-count. The lack of consistent direction in disagreement with manually counted steps for individuals makes it difficult to predict or account for error in these devices. Of the commercially available devices, the Garmin Vivofit 4 and Yamax SW200 demonstrated best agreement with manually counted steps. They also had the smallest range of error, albeit 197.2 and 173.2%. However, the limits of agreement were wide, particularly when compared to the mean total step-count. During a period of activity that results in a mean of 443 steps, the Garmin Vivofit 4 may overestimate steps by up to 756 steps and underestimate steps by up to 254 steps.

Of the three devices that provided activity minutes, it was not clear from the device manuals if activity minutes related to minutes in LPA, MVPA, LPA, and MVPA combined, or some other quantity. We, therefore, compared activity minutes from each device to LPA, MVPA, and total activity. However, activity minutes from all devices were not a good estimate of any measure of PA. Although the Letscom demonstrated the smallest median error compared to both total activity and LPA, the percentage error ranged from 5.6 to 65.1% for total activity and 5.3 to 63.3% for LPA. There was no evidence that activity minutes from the Fitbit Alta differed to minutes in MVPA. However, the Fitbit Alta recorded 0 min for 17 of the 19 participants, when only six participants had 0 min in MVPA, and none had 0 min in LPA or total activity. In addition, one of the two participants that the Fitbit Alta recorded > 0 activity minutes for did, in fact, have 0 min in MVPA. Overall, these data suggest none of these monitors should be used to estimate time in activity for people with MS.

No device was clearly preferred by the participants. However, four basic requirements were identified as being important for acceptability. These were being wrist worn (based on easier attachment and visibility), clear display, perceived accuracy, and offering something more than just step count. The latter is rooted in personal preference, with some participants preferring basic functionality, while others wanting more advanced monitoring features. Therefore, optional add-ons should be available to enhance acceptability for all users. Similarly, although aesthetics was important to many participants, requirements in terms of aesthetics differed between the participants.

Letscom was a clear favorite in terms of display characteristics and ease of data retrieval. The Garmin Vivofit 4 elicited the most negative reactions when the participants shared their thoughts on the device interface, as well as identifying difficulties synching the device. Similar issues were previously identified when exploring adults with MS experiences of monitoring step-count using the Yamax SW200 (18). However, this study highlights that these issues are not unique to the Yamax SW200. The strong emphasis on ease of attachment and charging and the visual display directly relate to additional physical challenges that people with MS may experience (23), and strongly indicate the need for manufacturers to consider the accessibility of their devices for people with impairments. Impaired vision and manual dexterity are not unique to MS and, in fact, may be experienced by many people as they age. Manufacturers need to consider designing products that are accessible to all. This has potential benefits to all users and not just those with specific conditions.

Perceived accuracy of the device was the overriding integral element to acceptability among people with MS. The participants particularly perceived the Yamax SW200, Letscom, and Garmin Vivofit 4 as inaccurate, despite the Garmin Vivofit 4 and the Yamax SW200 demonstrating best agreement with manually counted steps. We similarly identified that people with MS perceived the Yamax SW200 to be inaccurate when using it to monitor step-count over 12 weeks (18). This perception may be partly caused by these monitors recording steps during inactivity, which would have been particularly noticeable to participants when monitoring their activity at home. We found that the Yamax SW200 and Garmin Vivofit 4 incorrectly recorded steps during deskwork for 36.8 and 31.6% of the participants and for 100% of the participants during driving. The Letscom incorrectly recorded steps during deskwork but for a smaller proportion of the participants and for fewer participants than the Fitbit Alta did. However, the Letscom showed the largest percentage error, biggest range of error, and 95% of the participants had an error ≥25%. The Letscom also showed particularly poor agreement with manual step-count in terms of the limits of agreement. The limits of agreement were only wider for the Fitbit Alta. The large variability in error for individuals may explain why some participants commented on the Letscom being particularly inaccurate.

In agreement with previous findings that the objective numeric feedback provided by these devices can be a powerful motivational tool for some people with MS (18), the role of the devices as motivators was voiced in this study. This supports the potential use of wearable devices in physical activity behavior change interventions. However, the results of this study suggest that these commercially available devices may not provide valid estimates of step-count for adults with MS. Perceived inaccuracies of devices cast doubt on the value of them as monitoring tools and lead to frustration, distrust, and negatively affect people's motivation to use them (18).

Physical inactivity is a major global health concern for people with MS (24), with many people with MS spending two times as long seated compared to the general population (25). There is also a suggestion that improved physical activity levels among people with MS are indirectly associated with improvement in quality of life (26). As a result, key clinical guidelines specifically recommend tackling a lack of physical activity through targeted behavior change interventions (27). Activity monitors and pedometers are often used as an adjunct for such interventions and have been shown to increase motivation in home-based programmes, promoting increases in physical activity (18). However, it is pertinent that the validity and the acceptability of such devices are determined to aid device selection, especially for use among people with MS for whom 85% report gait deficits as their main presenting complaint (3). Although this study was conducted in a controlled environment, the tasks were chosen to capture common activities of daily living, including outdoor walking and driving where possible. This helps with the transferability of results beyond the laboratory environment into real-life scenarios, which has not been examined before. Considering that many people with MS use mobility aids and experience gait deficits (12), examining validity during simulated real-life scenarios, including navigating obstacles, is particularly important. However, future research should examine the validity of commercially available devices in free-living settings. A challenge when evaluating such devices is that technology is rapidly developing, with many devices being discontinued and replaced by new devices in short periods of time. Although the Fitbit Zip is the most commonly assessed commercially available device (11), it has been discontinued since this study was conducted. This somewhat limits the findings of this study. However, the findings from this study should be used as overarching principles to consider when developing or updating devices. The findings are also limited to ambulatory adults with MS, and, therefore, any recommendations for improving the validity and acceptability of these devices are not inclusive of wheelchair users.

## Conclusion

In conclusion, commercially available devices demonstrated poor criterion validity when measuring step-count and time in activity among people with MS. This negatively affected the acceptability of devices, with perceived inaccuracies causing distrust and frustration. However, perceptions of how accurate devices varied between individuals, reflecting the large amount of variability in individual error observed during validation. Perceived accuracy was the overriding integral element to acceptability of these devices among people with MS. However, additional considerations when designing devices for people with MS include an appropriately sized and lit display and ease of attaching and charging devices. These considerations would potentially improve the acceptability and inclusivity of devices for all and not just people with MS.

## Data Availability Statement

The datasets presented in this article are not readily available because consent was not obtained from participants to share data. Requests to access the datasets should be directed to meriel.norris@brunel.ac.uk.

## Ethics Statement

The studies involving human participants were reviewed and approved by Ethical approval was provided by Brunel University London's Research Ethics Committee (REC) and the Health Research Authority and Health and Care Research West Scotland (REC reference 18/WS/0161). The patients/participants provided their written informed consent to participate in this study.

## Author Contributions

GL, JFl, and JH completed data collection. JB and HJ contributed to data acquisition. JR, MN, and GL completed analysis and drafted the manuscript. JR, MN, GL, JFo, AS, and CK contributed to conception and design of the work. All authors contributed to the article and approved the submitted version.

## Funding

This study was supported by funding from RCSI University of Medicine and Health Sciences STAR Programme and Brunel University London.

## Conflict of Interest

The authors declare that the research was conducted in the absence of any commercial or financial relationships that could be construed as a potential conflict of interest.

## Publisher's Note

All claims expressed in this article are solely those of the authors and do not necessarily represent those of their affiliated organizations, or those of the publisher, the editors and the reviewers. Any product that may be evaluated in this article, or claim that may be made by its manufacturer, is not guaranteed or endorsed by the publisher.
