# Supplementary material for: Validity and Acceptability of Wearable Devices for Monitoring Step-Count and Activity Minutes Among People With Multiple Sclerosis
Source: Front Rehabil Sci. 2022 Jan 11;2:737384. doi: 10.3389/fresc.2021.737384 (PMC9397948; doi:10.3389/fresc.2021.737384)
Supplement: Supplementary file 1 [file Table_1.docx]

| **Name** | **Approximate price (2017)** | **Monitors step count** | **Monitors physical activity** | **Displays on watch** | **Attachment** | **Has clock function** | **Prompts to move** | **Battery** |
| --- | --- | --- | --- | --- | --- | --- | --- | --- |
| Fitbit Alta | £100 | Yes | Distance, calories, active minutes, hourly activity | Yes | Watch | Yes | Yes | Charge (5 days) |
| Fitbit Zip | £50 | Yes | Distance, calories | Yes | Clip on. No cover | Yes | No | Replaceable battery; lasts up to 6 months |
| Garmin Vivofit 4 | £70 | Yes | Distance, calories, activity | Yes | Watch | Yes | No | Replaceable battery; lasts up to 1 year |
| Yamax SW200 Digi-walker Pedometer | £19.75 | yes | No | Yes | Clip on | No | No | Replaceable battery |
| LETSCOM Fitness tracker | £20-30 | steps | Active minutes, distance, calories | Yes | Watch | Yes | No | Charge (7 days) |

Supplementary Table 1 Characteristics of Selected Commercially Available Wearable Devices
